# The Sense of Resilience of health care professionals in Latvia measured by Antonovsky’s Sense of Coherence Scale

**DOI:** 10.1192/j.eurpsy.2024.1189

**Published:** 2024-08-27

**Authors:** D. Janovskis, R. Eglītis

**Affiliations:** ^1^Department of Medicine, MD Resident; ^2^Department of Psychology, Mag. Psych., LU (Latvian University), Riga, Latvia

## Abstract

**Introduction:**

In this research work the sense of resilience was studied, which is an essential factor in reducing the stress of health care workers, it also helps to maintain the sustainability of the work of medical specialists and prevents the burnout syndrome. Aaron Antonovsky’s Sense of Coherence scale was used in this study, which helps to determine how health care professionals are able to preserve their mental and physical health.

**Objectives:**

This study is aimed to determine the differences in components of Antonovsky’s Sense of Coherence scale for various health care specialists and to describe the factor structure of Coherence scale for health care specialists in Latvia, that forms the sense of resilience for health care specialists.

**Methods:**

The questionnaire used in the study is Antonovsky’s Sense of Coherence scale’s (Antonovsky, 1987) Latvian version, that was translated into Latvian and adapted in the research work of A. Veylande, N. Bahmačova (2000). 202 respondents who are representatives of medical professions took part in this study. The obtained data were entered into the MS Excel computer program and were statistically processed using the SPSS 22 computer program.

**Results:**

Looking at the obtained results of this study, it can be stated that Medical Doctors- Specialists have statistically significantly higher Comprehensibility scores than Medical Orderlies (p = 0.01, r = 0.24), while Medical Doctors- Specialists and Medical Doctors- Residents have statistically significantly higher Manageability scores than Medical Orderlies (p= 0.04, r=0.21). Based on the analysis of the results, it has been determined that Doctors-Specialists (p=0.00, r=0.32) and Doctors-Residents (p=0.00, r=0.34) have statistically significantly higher Meaningfulness indicators than Medical Orderlies, as well as Doctors-Specialists (p=0.00, r=0.29) and Doctors-Residents (p=0.00, r=0.31) have statistically significantly higher Meaningfulness scores than Medical Nurses.

**Conclusions:**

Higher scores of the three components of Antonovsky’s scale for Medical Doctors-Specialists and Medical Doctors-Residents compared to Medical Orderlies and Medical Nurses could be explained by a lower overall time that Doctors spend in the department with patients. Higher education helps to overcome stress at work and to become aware of possible strategies for improving the joy of life, ways to reduce stress at work and to relax from work.

**Disclosure of Interest:**

None Declared

